# An optimized protocol for cytogenetics in stingless bees (Hymenoptera, Apidae, Meliponini)

**DOI:** 10.3897/compcytogen.20.218686

**Published:** 2026-10-30

**Authors:** Weslei Santos Nascimento, Beatriz Mafra Louzada, Júlia Alves Britto Peixoto, Ana Maria Waldschmidt

**Affiliations:** 1 Laboratory of Molecular Entomology, Department of Biological Sciences, Universidade Estadual do Sudoeste da Bahia, Jequié, Bahia, Brazil Laboratory of Molecular Entomology, Department of Biological Sciences, Universidade Estadual do Sudoeste da Bahia Jequié, Bahia Brazil

**Keywords:** Chromosome preparation, chromosome spreading, cell suspension, karyotyping, metaphase chromosomes

## Abstract

Cytogenetic studies in stingless bees date back to 1948 and have been useful to discriminate cryptic species and to infer the karyotype evolution in this diverse group of insects. However, a limitation of existing chromosome preparation protocols for bees is the lack of a cell suspension that can be stored for subsequent chromosome preparations. Therefore, the goal of this novel protocol was to obtain a suspension of dividing cells for each specimen allowing the analysis of an increased number of metaphase spreads per sample. This procedure was successfully applied to six species of Meliponini (*Melipona marginata* Lepeletier, 1836, *M. mondury* Smith, 1863, *M. scutellaris* Latreille, 1811, *Frieseomelitta flavicornis* Fabricius, 1798 (black morph), *Scaptotrigona* sp., and *Tetragonisca angustula* Latreille, 1811), with the potential to yield up to six slides containing well-spread metaphase chromosomes per larva. Thus, this protocol may optimize the use of collected larvae by providing cell suspensions that can be stored for subsequent cytogenetic analyses and potentially adapted for other hymenopterans.

## Introduction

Stingless bees (tribe Meliponini) have a pantropical distribution, playing a key role in the pollination of natural habitats and crops worldwide (Bueno et al. 2023). In spite of their relevance, the actual diversity of the stingless bees remains largely unknown, since there is considerable variation in the estimated number of species and genera in this group. For instance, Camargo et al. (2023) reported 412 species distributed among 33 genera of stingless bees, whereas Engel et al. (2023) recognized 605 extant species from 45 genera. Additionally, some authors recorded 37 genera, 17 of them encompassing at least 10 species each (e.g., Melo 2020). Only in Brazil, 259 species have been described from 28 genera (Nogueira 2023). This high diversity reflects the richness and complexity of these insects and highlights the challenges in resolving their taxonomic and systematic uncertainties.

On the other hand, cytogenetic studies in stingless bees have proved to be useful to discriminate species and populations, including the identification of cryptic forms (e.g., Barbosa et al. 2021; Campos et al. 2024; Cunha et al. 2024). In addition, these studies provided new insights into the chromosomal evolution in Meliponini, particularly when combined with molecular cytogenetics (Pereira et al. 2021; Campos et al. 2024). However, a major shortfall for the development of cytogenetics of insects is the obtaining of stored cell suspensions. Most techniques (see Imai et al. 1988) yield a single or a few slides per specimen, limiting the number of available metaphase spreads. In contrast, cell suspension obtained from a single larva can be distributed across multiple slides, allowing additional preparations from the same specimen. This is particularly relevant because analyses of several individuals are essential to confirm chromosome numbers, evaluate intraspecific variation, identify B chromosomes, and characterize heterochromatin patterns and ribosomal cistron localization. While careful sampling of brood combs is generally performed without significantly compromising colonies, using fewer larvae per colony may also reduce sampling impacts and handling-associated risks, including susceptibility to natural enemies such as phorid flies (Carvalho-Zilse et al. 2011; Pereira et al. 2012).

Therefore, a novel protocol, adapted from Imai et al. (1988), was developed to obtain a suspension of dividing cells from a single larva that can be stored for subsequent chromosome preparations. This approach allows fresh slides to be prepared when needed, including for different cytogenetic staining and banding techniques, potentially enabling multiple analyses to be performed using material obtained from the same larva, it may reduce the number of larvae required per colony and potentially minimizes the sampling impacts over small colonies. On the other hand, the long-term storage of cell suspensions from a single larva allows researchers to apply different cytogenetic techniques to the same individual at different times, potentially expanding the range of analyses that can be performed, such as taxonomic, systematic and evolutionary studies of stingless bees.

## Methods

Six species of Meliponini were chosen to demonstrate the applicability of the new protocol: three *Melipona* species (*Melipona marginata* Lepeletier, 1836, *Melipona mondury* Smith, 1863 and *Melipona scutellaris* Latreille, 1811) and three non-*Melipona* species (*Frieseomelitta flavicornis* Fabricius, 1798 (black morph); *Scaptotrigona* sp.; and *Tetragonisca angustula* Latreille, 1811) (Table 1).

**Table 1.** Stingless bee species analyzed using the novel protocol (locality, metaphases/slide, colchicine treatment (T), diploid number (2n)).

**Species**	**Collection site (state of Bahia, Brazil)**	Mean number of metaphases*	**T**	**2n**
*Melipona marginata*	Nova Ibiá	15.6	10 min	18
*Melipona mondury*	Nova Ibiá	30.0	10 min	8
*Melipona scutellaris*	Nova Ibiá	13.7	10 min	18
*Frieseomelitta flavicornis* (black morph)	Jequié	17.3	40 min	30
*Scaptotrigona* sp.	Nova Ibiá	15.0	50 min	34
*Tetragonisca angustula*	Jequié	5.8	37 min	34

*see detailed values in Suppl. material 1: table SS1.

The chromosomal preparations were performed as follows:

1. The cerebral ganglia were dissected from the post-defecating larvae in 0.005% hypotonic colchicine solution (sodium citrate 1%/colchicine 0.1%);
2. The extracted ganglia were added to 1 mL of 0.005% hypotonic colchicine solution (sodium citrate 1%/colchicine 0.1%) as described by Imai et al. (1988);
3. After defining the species-specific incubation period (see Table 1), the ganglia were transferred to an Eppendorf tube containing 1 mL of fixative I (3:3:4; 30% glacial acetic acid, 30% ethanol, and 40% distilled water);
4. The ganglia were then macerated using a pestle for approximately 1–2 minutes until a homogeneous suspension was obtained. The suspension can be stored at - 20 °C or proceed to step 5;
5. Heat clean glass slides at 60 °C in a water bath for 10 minutes;
6. Drop three droplets (about 0.05 mL) of the cell suspension onto a dry, pre-heated glass slide;
7. Add some drops of fixative II (1:1 50% ethanol/50% glacial acetic acid);
8. Keep the slides vertically on the laboratory bench to drain the excess of fixative;
9. Add three additional droplets of acetic acid;
10. Keep the slides vertically on the laboratory bench to drain the excess of fixative;
11. After air-drying, stain the slides in a 5% Giemsa solution in phosphate buffer (pH 6.8) for 12 min and then rinse the slides under running tap water.

The chromosome preparation protocol used in this study is available at protocols.io (https://doi.org/10.17504/protocols.io.4egnxbn6ql5d/v1).

The cell suspension from each sample can potentially be used to prepare up to six slides per larva. However, the quantity and overall condition of metaphase chromosome spreads varied among specimens and species. In particular, *Tetragonisca angustula*, the smallest species analyzed, showed a lower abundance of metaphases, suggesting that body size and the amount of available biological material may influence the number of slides that can be effectively prepared and analyzed. Thus, the number of slides may need to be adjusted according to species, with fewer slides potentially being more appropriate for smaller bees and other species-specific methodological adjustments being necessary to achieve optimal results. Nonetheless, the use of this protocol allowed the preparation and visualization of metaphase chromosomes in all species evaluated in this study, supporting its potential applicability across the species analyzed.

Furthermore, to assess the potential applicability of the chromosome preparations to further banding techniques, fluorescence *in situ* hybridization (FISH) was performed to map the (GA)_15_ microsatellite and 18S rDNA. The (GA)_15_ microsatellite was mapped using a synthetic oligonucleotide directly labeled with Cy3 at the 5' end (Sigma-Aldrich), following the protocol of Pinkel et al. (1986), with modifications. Chromosomal DNA was denatured in 70% formamide/2×SSC at 72 °C for 5 min, while the hybridization mixture containing the probe was denatured at 85 °C for 10 min. The mixture was then applied to the slides and incubated overnight at 37 °C in a humid chamber. The 18S rDNA probe was labeled with biotin-14-dATP using the BioNick Labeling System (Invitrogen), and the hybridization signals were detected using FITC and avidin conjugated to FITC. After post-hybridization washes, the preparations were counterstained and mounted with 4',6-diamidino-2-phenylindole (DAPI) in Fluoroshield (Sigma-Aldrich) (see Suppl. material 1: figs S1, S2). No evidence of chromosomal changes or degradation was observed following the procedures.

## Results

The diploid chromosome numbers observed in all species analyzed in this study are consistent with values reported in the literature: *Melipona* Illiger, 1806 (2n = 18), *Scaptotrigona* Moure, 1942 and *Tetragonisca* Moure, 1946 (2n = 34), and *Frieseomelitta* Ihering, 1912 (2n = 30) (Kerr 1948; Duarte et al. 2009; Barth et al. 2011; Santos et al. 2018; Cunha et al. 2024) (Table 1; Fig. 1). The optimal colchicine exposure time varied considerably among stingless bee species, ranging from 10 minutes (*Melipona*) to 50 minutes (*Frieseomelitta*). Metaphase chromosomes were generally suitable for visualization, although chromosome spreading and cellular debris varied among preparations. The use of a heated water bath during slide preparation may contribute to chromosome spreading, consistent with previous reports describing heating as a strategy for improving metaphase chromosome spreading (Deng et al. 2003).

**Figure 1.**
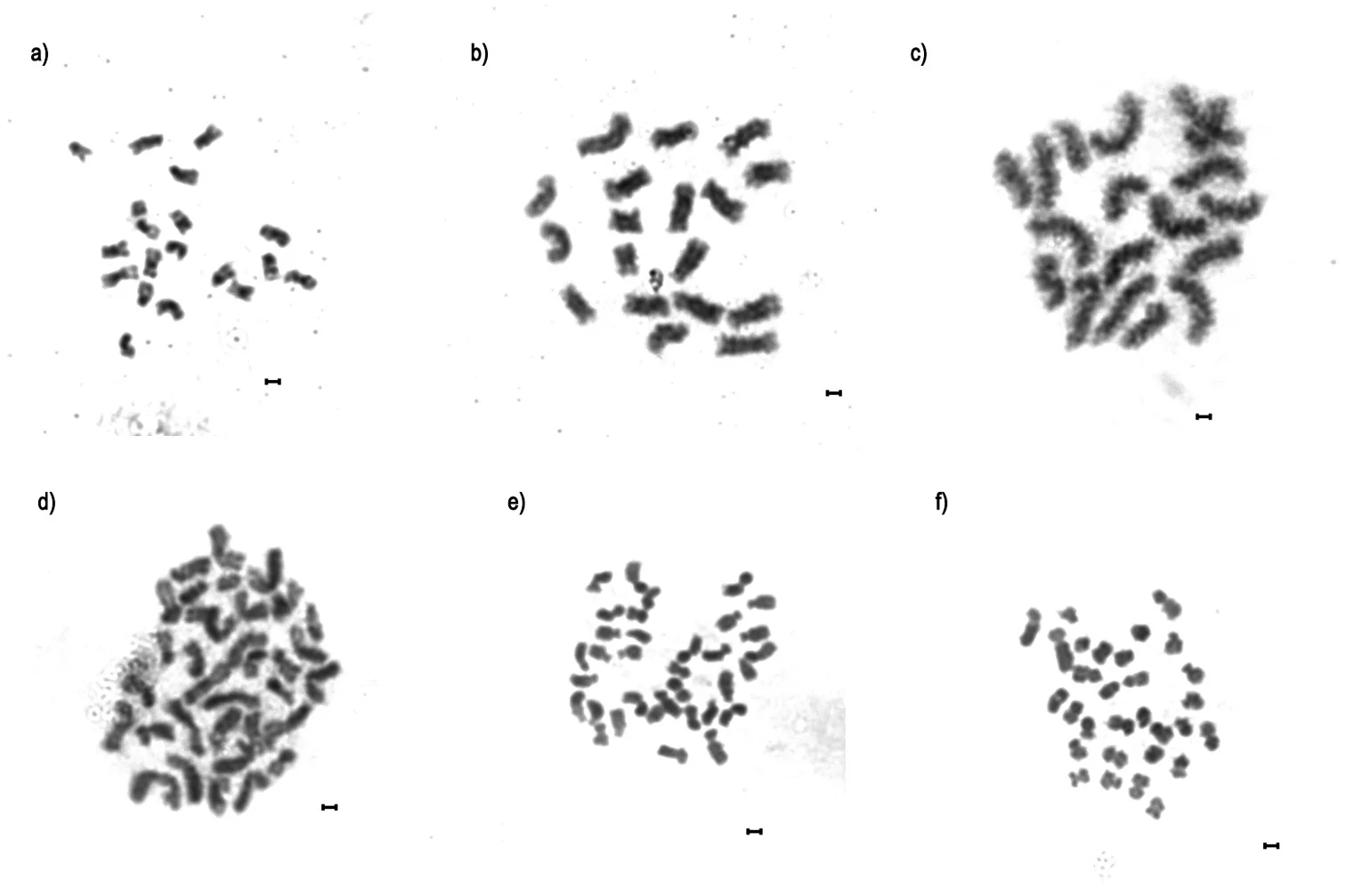
Metaphase spreads obtained using the novel protocol for cytogenetic studies in stingless bees: **a**. *Melipona marginata*, **b**. *Melipona mondury*, **c**. *Melipona scutellaris*, **d**. *Tetragonisca angustula*, **e**. *Scaptotrigona* sp., **f**. *Frieseomelitta flavicornis* (black morph). Scale bar: 10 µm.

## Discussion

Although an increasing number of cytogenetic studies have been conducted on stingless bees, substantial gaps in cytogenetic knowledge remain, and these studies have provided valuable insights into their diversity patterns. For example, B chromosomes have been reported in *Tetragonisca fiebrigi* (Schwarz, 1938) and can serve as cytotaxonomic markers to distinguish this species from *Tetragonisca angustula* (Barth et al. 2011). Differences in karyotypic traits have also been observed among populations of the same taxon, indicating population structure that should be considered in the management and conservation of native species (Duarte et al. 2009; Campos et al. 2024). More recently, microsatellite and rDNA mapping has contributed to our understanding of the ancestral chromosome number and chromosomal evolution in Meliponini (Cunha et al. 2024).

Despite the description of new species (Nogueira et al. 2022; Ribeiro et al. 2024), cytogenetic data are still lacking for many taxa, limiting comparisons that could help clarify species boundaries and evolutionary relationships. Taxa traditionally studied using procedures based on Imai et al. (1988) may also benefit from the protocol presented here, although further studies are needed to determine how widely it can be applied. In addition, storing the cell suspension allows further chromosome slides to be prepared when needed, making it possible to obtain more slides from the same larval material and use the collected biological material more efficiently.

## Conclusion

In conclusion, this protocol offers practical advantages for cytogenetic studies of stingless bees. By obtaining a suspension of dividing cells from a single larva for long-term storage, researchers can apply different cytogenetic techniques to the same individual at any time. These features might optimize the cytogenetic analyses of a single sample of stingless bee species, particularly in relation to the application of distinct banding methods per individual while also providing a basis for potential adaptation of the protocol to other hymenopterans.

## Additional information

### Artificial Intelligence (AI) use

The authors accept full responsibility for the content of the manuscript, including the disclosure of any use of AI.

No AI tools were used in the preparation of this manuscript.

### Author ORCIDs

W.S. Nascimento https://orcid.org/0009-0009-0167-3139

B.M. Louzada https://orcid.org/0009-0006-6357-4329

J.A.B. Peixoto https://orcid.org/0009-0008-4249-5693

A.M. Waldschmidt https://orcid.org/0000-0002-6366-9954

### Data availability

All of the data that support the findings of this study are available in the main text or Supplementary Information.

## Supplementary materials

Supplementary material 1 Supplementary table and figures Data type docx Explanation note **table SS1**. Number of slides prepared and metaphases observed per slide for each species and individual; **figure S1**. Metaphase of *Melipona mondury*; **figure S2**. Metaphase of *Scaptotrigona* sp. https://binary.pensoft.net/file/1825254This dataset is made available under the Open Database License (http://opendatacommons.org/licenses/odbl/1.0/). The Open Database License (ODbL) is a license agreement intended to allow users to freely share, modify, and use this Dataset while maintaining this same freedom for others, provided that the original source and author(s) are credited. Weslei Santos Nascimento, Beatriz Mafra Louzada, Júlia Alves Britto Peixoto, Ana Maria Waldschmidt
