# Supplementary material for: An optimized protocol for cytogenetics in stingless bees (Hymenoptera, Apidae, Meliponini)
Source: Comp Cytogenet. 2026 Oct 30;20:245–52. doi: 10.3897/compcytogen.20.218686 (PMC13743983; doi:10.3897/compcytogen.20.218686)
Supplement: Supplementary file 1 [file comparative_cytogenetics-20-245_article-218686__-s001.docx]

**Supplemental material**

An optimized protocol for cytogenetics in stingless bees (Hymenoptera: Apidae: Meliponini)

Weslei Santos Nascimento, Beatriz Mafra Louzada, Júlia Alves Britto Peixoto and Ana Maria Waldschmidt

Laboratory of Molecular Entomology, Department of Biological Sciences, Universidade Estadual do Sudoeste da Bahia, Jequié, Bahia, Brazil.

Corresponding author: Weslei Santos Nascimento (wesleisn20@gmail.com)

Orcid ID - Weslei Santos Nascimento (https://orcid.org/0009-0009-0167-3139), Beatriz Mafra Louzada (https://orcid.org/0009-0006-6357-4329), Júlia Alves Britto Peixoto (https://orcid.org/0009-0008-4249-5693) and Ana Maria Waldschmidt (https://orcid.org/0000-0002-6366-9954)

**Table S1.** Number of slides prepared and metaphases observed per slide for each species and individual.

| Species | Individuals* | Number of slides** | Number of metaphases*** |
| --- | --- | --- | --- |
| *Melipona marginata* | 1  2  3 | 2  2  1 | Slide 1: 10; Slide 2: 10  Slide 1: 10; Slide 2: 25  Slide 1: 23 |
| *Melipona mondury* | 1 | 2 | Slide 1: 25; Slide 2: 35 |
| *Melipona scutellaris* | 1  2 | 2  1 | Slide 1: 6 ; Slide 2: 5  Slide 1: 30 |
| *Frieseomelitta flavicornis*  *(*black morph*)* | 1  2  3 | 2  3  3 | Slide 1: 3; Slide 2: 6  Slide 1: 20; Slide 2: 25; Slide 3: 8  Slide 1: 20; Slide 2: 40; Slide 3: 16 |
| *Scaptotrigona* sp*.* | 1  2 | 1  1 | Slide 1: 20  Slide 1: 10 |
| *Tetragonisca angustula* | 1  2  3 | 1  2  2 | Slide 1: 8  Slide 1: 2; Slide 2: 6  Slide 1: 3; Slide 2: 10 |

* Larvae used for cytogenetic preparation; each number represents one individual.

** Number of slides prepared from each individual.

*** Number of metaphases observed on each slide

**Figure S1.** Metaphase of *Melipona mondury*. **A** 4′,6:diamidino:2:phenylindole (DAPI) staining. **B** (GA)₁₅ probe signals detected with Cy3. **C** merged images. Scale bar: 10 µm.

**Figure S2.** Metaphase of *Scaptotrigona* sp.. **A** 4′,6:diamidino:2:phenylindole (DAPI) staining. **B** 18S rDNA probe signals detected with FITC. **C** merged images. Scale bar: 10 µm.
